# An Incident of Practice

**Published:** 1882-10

**Authors:** C. E. Francis

**Affiliations:** 33 W. Forty-seventh St.; N. Y. City


ARTICLE II.

### AN INCIDENT OF PRACTICE.

C. E. FRANCIS, D. D. S., M. D. S., N. Y. CITY.

A young gentleman, a theological student, recently called for advice and treatment. About two weeks before, a troublesome inferior first molar had been removed, but a portion of a root remained. His face, which he said was then badly swollen, was still in that condition. The muscles of the cheek were exceedingly rigid, and it was quite difficult to get his mouth sufficiently open for examination. Pus had formed, which had worked its way into the cheek almost to the outer surface, whither it had collected and threatened to perforate the skin.

The inflammation extended down his neck, and the sub-maxillary gland was considerably enlarged.

The patient was of scrofulous diathesis, possessing a delicate constitution with an apparent tendency to phthisis.

Just before coming to me he had been under medical treatment. His physician had treated his face to warm poultices, and had also made an effort to reach the abscess by lancing the cheek from the inside, but failing to do so, abandoned his efforts, saying it was impossible to prevent the pus from coming through the face.

At the suggestion of a friend he called on me. Finding him in a constipated condition, I advised small teaspoonful doses of Rochelle salts hourly until a free movement of the bowels was obtained, also a small dose each day, to keep them free. Directed him to apply a mixture of equal parts of Tr. Iodini Comp., Tr. Opii., and Glycerine twice a day. Procured for him a saucer-shaped piece of sheet lead about four inches in diameter, requesting him to line the concave surface with several thicknesses of old linen kept wet with Fl. Ext. Hamamelis—this wet compress to apply to his cheek and held in position with a light bandage.

That nothing might be left undone, I also advised the patient to keep, for a time, a piece of wasted fig between the cheek and gum, in hopes to invite the pus in that direction. Two days afterward he called. The inflammation had received a check and was abating. Directed the patient to take four grains Potassai Iodid., three times a day.

Two days later marked a decided improvement, and in a week from his first visit the inflammation had completely subsided, the muscles of the face were relaxed and the trouble ended. The pus had been carried off by absorbents, no opening having appeared inside or out.

I have in the course of my practice treated a number of similar cases in like manner and with successful results. Have known repeated instances where alveolar abscesses had coursed their way through the cheek, induced thither by hot poultices.

There is no necessity for an alveolar abscess to open through the cheek unless maltreated or too long neglected. Such openings are apt to leave a deep unsightly scar, which is a life long blemish.

33 *W. Forty-seventh St.*

